# Neural Mechanisms of Visual Motion Anomalies in Autism: A Two-Decade Update and Novel Aetiology

**DOI:** 10.3389/fnins.2021.756841

**Published:** 2021-11-01

**Authors:** Samuel Spiteri, David Crewther

**Affiliations:** Centre for Human Psychopharmacology, Swinburne University of Technology, Melbourne, VIC, Australia

**Keywords:** autism, ASD, visual area hMT+, pulvinar, visual perception, amygdala, dorsal stream

## Abstract

The 21st century has seen dramatic changes in our understanding of the visual physio-perceptual anomalies of autism and also in the structure and development of the primate visual system. This review covers the past 20 years of research into motion perceptual/dorsal stream anomalies in autism, as well as new understanding of the development of primate vision. The convergence of this literature allows a novel developmental hypothesis to explain the physiological and perceptual differences of the broad autistic spectrum. Central to these observations is the development of motion areas MT+, the seat of the dorsal cortical stream, central area of pre-attentional processing as well as being an anchor of binocular vision for 3D action. Such development normally occurs *via* a transfer of thalamic drive from the inferior pulvinar → MT to the anatomically stronger but later-developing LGN → V1 → MT connection. We propose that autistic variation arises from a slowing in the normal developmental attenuation of the pulvinar → MT pathway. We suggest that this is caused by a hyperactive amygdala → thalamic reticular nucleus circuit increasing activity in the PIm → MT *via* response gain modulation of the pulvinar and hence altering synaptic competition in area MT. We explore the probable timing of transfer in dominance of human MT from pulvinar to LGN/V1 driving circuitry and discuss the implications of the main hypothesis.

## Visual Perceptual Anomalies in Autism - A Turn of the Century View

It is over 15 years since Dakin and Frith (2005) published their seminal review on the visual perceptual anomalies associated with autism spectrum disorders (ASD). During this period, the reported prevalence of ASD has more than doubled (Maenner et al., 2020), over 100 genes strongly associated with autism have been reported (Satterstrom et al., 2020), the number of publications per year (PubMed) has grown from under 1000 to over 6000, yet fundamental cause, as well as effective treatment, are still lacking. However, hypo- and hyper-reactivity to sensory stimuli (American Psychiatric Association, 2013) now is recognised as one the most commonly occurring features, prevalent in over 90% of autistic children (Leekam et al., 2007), exacerbating (Thye et al., 2018) or perhaps feeding the core social difficulties experienced (Simmons et al., 2009).

As with many other neurodevelopmental disorders such as dyslexia, dyspraxia, Williams syndrome, amongst others, those with clinically defined ASD and also those with high trait levels of autism - as measured by the Autism spectrum Quotient (AQ) (Baron-Cohen et al., 2001) show a “dorsal stream vulnerability” (Braddick et al., 2003) as evidenced by raised motion coherence thresholds (Spencer et al., 2000; Milne et al., 2002; Braddick et al., 2003). Primate cortex shows a clear separation of its ventral and dorsal visual processing streams, the “what” and the “where” streams (Ungerleider and Mishkin, 1982) or “vision for perception” versus “vision for action” (Milner and Goodale, 2006). These ventral and dorsal stream networks are relatively independent (Fox et al., 2006) - damage associated with regions of the ventral stream do not severely impact the abilities of the dorsal stream and vice-versa (Goodale, 2013). The dorsal stream vulnerability in autism is typically exemplified by abnormalities in motion processing which, for ASD, Dakin and Frith (2005) attributed to aberrant processing in extrastriate cortical areas, particularly posterior superior temporal sulcus (pSTS). This was on the basis of the notion of normal early sensory processing by the M-pathway, citing lack of difference in flicker contrast sensitivity at 10 Hz modulation (Pellicano et al., 2005). In addition, normal first order (Bertone et al., 2003) but abnormal second order motion processing (Bertone et al., 2005) have been reported. Stimulus complexity was seen as being higher order and hence further along the visual pathway hierarchy. Moreover, the pSTS is seen as a centre for motion processing of socially relevant stimuli (Vaina et al., 2001) - with a clear connection to the behavioural and social symptoms of autism (Pelphrey et al., 2004; Hotier et al., 2017).

### Early Cognitive Theories of Perceptual Anomaly: Weak Central Coherence and Enhanced Perceptual Function

Two cognitive theories of perceptual abnormality emerged around the beginning of the 21st century – the weak central coherence (WCC) (Frith, 1992), updated (Happe and Frith, 2006), and the enhanced perceptual function (EPF) (Mottron and Burack, 2001) model. The WCC proposed that autistic children appeared to have either inability or difficulty on global processing tasks and would more veridically judge visual illusions compared to typically developing children. Mottron and Burack (2001) suggested that autism could be better explained by an overdeveloped low-level processing bias rather than a global processing deficit, beginning in early childhood, as the consequence of compensating cognitive deficits occurring from localised areas of the brain. Mottron et al. (2006) updated the EPF model, suggesting that local bias was a superiority of low-level perceptual operations, suggesting that the endophenotype of autism could be explained by the superior functioning, involvement, and autonomy of the posterior and central parts of the visual cortex.

### Theories Without Mechanisms

Of course, motion processing is not the only perceptual discriminant of autism compared with typically developing perception. Shah and Frith (1983) pointed to a superior ability of those with the condition to observe fine/static detail, an observation often repeated. This transformed into a theoretical description ascribing a relative bias for local rather than global information processing (O’Riordan and Plaisted, 2001; Kaldy et al., 2016; Van der Hallen et al., 2019; Constable et al., 2020) with similar results reported across the autistic phenotype (Cribb et al., 2016; English et al., 2017; van Boxtel et al., 2017; Burling et al., 2018) employing high and low scoring cohorts on the AQ scale in its adult (Baron-Cohen et al., 2001) adolescent (Baron-Cohen et al., 2006) and child (Auyeung et al., 2008) forms.

In addition, aspects of rigidity of autistic behaviours has been incorporated into Bayesian models (Pellicano and Burr, 2012) with the notion of “hypo-priors” in those with high autistic tendency resulting in more veridical perception. Another aspect of autistic behaviour – recognised in early work by Kanner (1943) was the hyper-sensitivity often expressed by autistic children. With this range of perceptual anomalies, it is unsurprising that perceptual learning is over-specified in autism (Harris et al., 2015).

Of course, the specification of autistic anomalies and the descriptive models help enormously in trying to understand the behaviours associated with autism. An important search is for neural mechanisms, particularly developmentally sensitive ones, that result in prediction of autistic behaviours. Hence, the changes in understanding of primate visual development that have occurred over the past couple of decades need to be incorporated into the set of autistic mechanisms.

## Updating Primate Developmental Visual Neuroscience

Over the past two decades, understanding of primate neuroanatomy and neurophysiology has undergone a striking change – particularly in terms of neural development. As a highly informative representative review from that earlier era, Nassi and Callaway (2009) clearly laid out the projections of the major retinal ganglion cell (RGC) types, with their photoreceptoral sources as well as termination patterns in the lateral geniculate nucleus (LGN) and their projections into primary visual cortex (V1) and onwards into the dorsal and ventral cortical streams. Thus, they described the LGN magnocellular (M) cells receiving input from the parasol class of RGC, the parvocellular (P) cells receiving input from midget RGCs, as well as the Koniocellular layers of the LGN receiving input from the bistratified RGC providing colour input along the Blue-Yellow colour coordinates as well as other low-population cell types. In terms of area V1 projections, the well-known layer IVCα, IVC β terminations of M and P neurons, respectively, are described as well as the terminations of koniocells in the blob regions of layer II/III. In addition, direct projections from the konio layers of the LGN to area MT+, bypassing area V1 are reported (Sincich et al., 2004), yielding a di-synaptic pathway from the retina → LGN → MT. Nassi and Callaway (2007) investigated specialised MT-projecting neurons in layer 4B of area V1 of the macaque, suggesting these (mainly) spiny stellate cells are specialised for fast transmission from the M pathway, while the pyramidal cell projections from layer 4B of area V1 to area V2 seem to involve mixed M and P signals, with slower computations.

**FIGURE 1:**
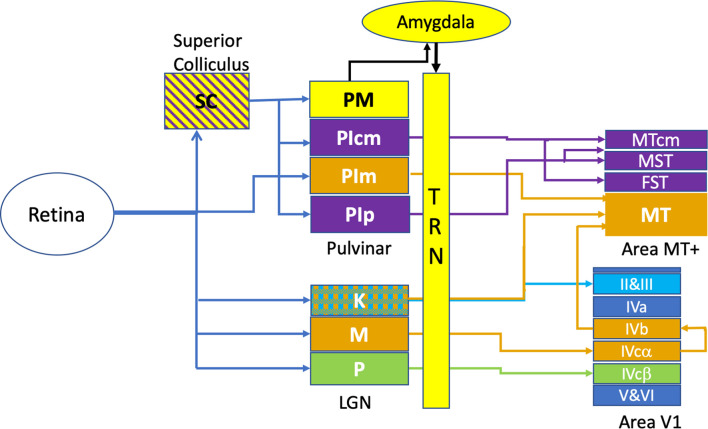
Retino-cortical circuits involved in rapid visual responses. This figure illustrates the most rapid connections between retina and area MT+, omitting many multi-synaptic connections. The colour coding of the diagram relates to the endpoint of transmission, with Area MT and its thalamic afferents shown in orange. In a similar fashion, the motion sensitive areas surrounding Area MT -namely MST, FST, MTcm and their afferents are labelled in purple. The Parvocellular inputs (P) to Area V1 are labelled in green while the koniocellular (K) laminae of the LGN are cross-hatched, indicating projections to both Area MT as well as to the superficial layers (II and III) of Area V1. Rapid emotional processing is indicated by the pathway from superior colliculus (SC) to the medial pulvinar (PM) and thence to the Amygdala which projects strongly to the thalamic reticular nucleus (TRN). This pathway, putatively provides emotional attention *via* thalamic response gain modulation, is coloured in yellow.

Advances in anatomical tracing techniques as well as the introduction of the marmoset, a new world primate, in post-natal developmental studies, have changed fundamentally our understanding of motion processing and the drivers of the dorsal cortical stream. These studies have shown that areas MT and V1 appear crucial in supporting the development of the dorsal and ventral cortical streams, respectively (Warner et al., 2012). The extra-striate cortical area MT, primarily involved in motion processing, stereopsis and transient attention receives mainly magnocellular derived inputs from area V1 as well as a small direct koniocellular input (from layers K1, K3 of the LGN) in the adult marmoset (Mundinano et al., 2019b), and is connected to various satellite areas such as the medial superior temporal area (MST) and fundus of the superior temporal sulcus (FST) (Kaas and Lyon, 2007; Zeki, 2015), cortical regions involved in visual pursuit. Recent anatomical studies in the marmoset point to a disynaptic pathway from retina to MT *via* the medial subregion of the inferior pulvinar (PI_m_). The surrounding PI subregions – PI_p_ and PI_cm_ receive input from the superior colliculus (SC) and project directly to MST and FST, and MTcm but not to area MT (Kwan et al., 2019). In terms of the quantitation of projections from these different sites, in the adult, V1-MT neurons comprised nearly 76% and PI_m_-MT neurons comprise 23% while direct LGN-MT are small in number – roughly 1% (see Figure 1).

The direct pulvinar → MT pathway is not served by the parasol RGC type (magnocellular) – rather it appears that the widefield ganglion cell class contributes this direct retina → PIm connection (Kwan et al., 2019). These cells are characterised by large dendritic fields and are perhaps 2–3 times the diameter of a parasol cell dendritic field at the same retinal eccentricity. While other small populations of large dendritic field ganglion cell exist, the broad thorny cell is one class that projects to the superior colliculus and has physiological properties consistent with utility in visual pursuit (Puller et al., 2015).

The pulvinar (PUL) is the largest thalamic nucleus, taking up ∼30% of its volume in humans and comprises four major divisions: the inferior (PI), lateral (PL), medial (PM), and anterior (PA) divisions (Arcaro et al., 2015). Although the pulvinar is known to have a multitude of connections and thought to possess a variety of functions, it is certainly involved in visual attention and salience, sharing relay connections to the Superior colliculus (SC) and direct connections to MT *via* the PI (Stepniewska et al., 1999; Kaas and Lyon, 2007; Berman and Wurtz, 2010; Warner et al., 2010; Benarroch, 2015), as well as direct connections to the amygdala (Elorette et al., 2018).

### Normal Visual Development in Primate

In a disruption to conventional views suggesting that LGN → V1 connections drive development of both cortical streams it now appears that the dorsal stream with its MT projections precedes development of the ventral stream. Marmoset studies demonstrate that although both MT and V1 emerge at similar times projections from the PUL → MT likely supports the development of the dorsal stream while V1 likely supports the development of the ventral stream (Mundinano et al., 2015). Additionally, early maturation of dorsal area MT was found to be influenced by di-synaptic retina → PI → MT connections instead of V1 or retinogeniculate (retina → LGN → MT) inputs (Warner et al., 2012). Dorsal stream associated areas were also found to emerge significantly earlier than ventral stream areas, for example after development of MT, its satellites (MTcm, MST, and FST) develop simultaneously, whereas ventral stream associated areas (V2, V3, V4, and the inferotemporal cortex) develop hierarchically after V1 (Mundinano et al., 2015). The emergence of an independent seed area for cortical development requires close neural communication and this is achieved by specialised reciprocal connections between layer 4B of V1 and layer 4 of MT/V5 (Shipp and Zeki, 1989; Nassi and Callaway, 2007) (in marmoset the corresponding connection is between Layer 3C of V1 to Layer 4 of MT) (Warner et al., 2012).

The functional development of each stream has shown a somewhat variable pattern in both human and non-human species. Some argue that when compared to the ventral stream, dorsal stream related areas mature later, are less activated and increase more in volume (Klaver et al., 2008; Loenneker et al., 2011; Smith et al., 2017) while others find an earlier dorsal stream maturational pattern, occurring at 4–5 (James and Kersey, 2018) and 6 years old (Ciesielski et al., 2019). Parallel development and maturation of both streams occur at somewhat similar times in macaques aged from around 1 month to 2 years (Van Grootel et al., 2017). By comparison, human visual maturation appears to occur from 3 to 12 years (Parrish et al., 2005; Braddick and Atkinson, 2011). Several factors are at play here. For example, the maturation of visual spatial acuity depends on the maturation of the photoreceptor outer segments (Hendrickson et al., 2012), eyeball axial length (affecting photoreceptor spacing, hence angular subtense), as well as cortical development and maturation including the processes of myelination.

In terms of developmental staging, it appears that certain divisions of the pulvinar and MT are vital for early visual development. Robust retinorecipient connections initially exist between the medial portion of the inferior pulvinar (PI_*m*_) and MT in the neonatal marmoset. This profuse PI_*m*_ → MT projection from the retina as well as from the SC to PI_*p*_, PI_*cm*_ → MTc, MST, FST is present around post-natal day (PD) 7–30 but is substantially pruned from PD 90, taken over by LGN → V1 → MT connections. In humans, a similar developmental pattern possibly emerges with strong retino-pulvinar → MT connections likely providing early visual inputs to infants, but it too undergoes a takeover from geniculo-striate connections, beginning to exert visual dominance approximately 2 months after birth (Braddick and Atkinson, 2011; Bridge et al., 2016).

The effects of neonatal damage to V1 on visual development is fascinating. Utilising diffusion magnetic resonance imaging (dMRI) Warner et al. (2015) showed that after lesioning V1 of a neonatal marmoset the retino-pulvinar → MT track (that normally withdraws) not only remained strong yielding a 60 times greater streamline number than control adults, while adult-lesioned marmosets were found to have a 38% reduction, when compared to their respective non-lesioned controls.

Furthermore, the neonatal V1 lesioned marmosets had increased pulvinar → MT tracts and were found to exhibit a preservation of conscious visual capacity. V1 lesions carried out in adults resulted in blindness. This phenomenon is beyond blindsight; a condition whereby occipitally lesioned individuals are able to respond to some visual stimuli (moving, low spatial frequency) without conscious perception (Bridge et al., 2016).

### Measurement of M and P Responses in Human

Early studies of the temporal properties of M and P neurons (Kaplan et al., 1990) revealed the different contrast response properties and temporal dynamics of single cell recordings in the LGN and the retina (Kaplan and Benardete, 2001). However, attributing certain functions and abnormalities to particular visual streams has required the suite of non-invasive techniques available – mainly psychophysical, with support from structural and functional MRI, EEG and to a lesser extent MEG. This was until it was realised that the non-linearities generated in the VEP by rapid stimulation had a physical significance (Klistorner et al., 1997).

### Wiener Kernel Analysis of VEP Non-linearities

In terms of temporal function, the visual system is highly non-linear. One has only to think of a fluorescent lamp: we cannot see it flicker but know that it does at twice the electrical mains frequency. On the other hand, we are always able to see the lamp turning on or off. One productive way of measuring the temporal structure of the visual system is *via* white noise analysis – i.e., using random stimuli. This has been further enhanced through the use of pseudo-random binary stimulus sequences where the input-output relations can be easily analysed through cross-correlation. Many of the attempts to measure non-linear physiological effects of stimulation have been based on the Volterra expansion (Volterra, 1959). Just as we approximate a function *f*(*x*) around the value *x* = a, given the value of *f* and its derivatives evaluated at a, by a Taylor series expansion, the Volterra series is like a Taylor series with time dependence – it approximates a system where the output depends on past inputs.

The Wiener kernel expansion as described by Sutter (1992) is similar except that in the power series approximation, the successive kernels are orthogonalised with respect to all those of lower order (Marmarelis and Marmarelis, 1978). The details of the Wiener kernel expansion as applied to visual evoked recording is laid out in a simple fashion by Sutter (2000).

Klistorner et al. (1997) showed, using diffuse flash VEP, that the non-linearities from the second order kernel tend to separate as a function of interaction time, ie across the slices of the second order kernel. Thus while a typical VEP (Ossenblok et al., 1994) shows variation in dominant peaks as a function of contrast, the first and second slices of the second order kernel (non-linear components measuring the effects of stimuli either one or two frames previous to the current frame, respectively) showed individual development of evoked responses with peak amplitudes showing characteristics for the K2.1 of high contrast gain (rapid increase in peak amplitude at low contrast) and saturation (semi-saturation at about 20% contrast) while the second slice K2.2 responses showed as a function of increasing contrast lower contrast gain and a lack of saturation at high contrast. In addition, comparison of the latencies of peaks of the K2.1 and K2.2 responses were shorter by about 20 ms for the K2.1 compared with K2.2. These characteristics conformed very well to the known contrast gain and saturation properties of the primate (Kaplan et al., 1990; Kaplan and Benardete, 2001). Another characteristic separating M and P neural function is in terms of temporal frequency response and it is clear that the flicker fusion threshold – the maximum frequency for achromatic flicker that can be perceived is a neuronal signature for M-pathway function (Benardete and Kaplan, 1999). Thus, a recent paper validates the identification of the major K2.1 wave with M-pathway function, showing that flicker fusion frequency in human (Brown et al., 2018) is negatively correlated with the amplitude of the second order slice K2.1. That is, the higher the frequency at which one can just distinguish flicker from a steady light, the smaller is the second order peak amplitude - introducing the idea of the non-linear VEP being able to measure neural efficiency.

### Development of M and P Function in TD Human

Braddick and Atkinson (2011) summarised the visual development of infants through to adult describing the increases in sensitivity to orientation, direction selectivity, motion sensitivity and binocular disparity. Maturation is variable across such quantities. Thus, while 2 month old infants are capable to determining direction selectivity – through preferential looking techniques, some other aspects of motion performance take much longer to mature. Thus, the sensitivity to global and biological motion does not reach adult levels until around the age of 14 years (Hadad et al., 2011). This is roughly the same age as reported by the Spekreijse lab for the maturation of the cortical VEP (Ossenblok et al., 1994). Although human synaptogenesis is most rapid from 2 to 4 months, synaptic elimination continues over a much longer period, from ages of roughly 8 months to 11 years in the visual cortex (Huttenlocher, 1990) which likely contributes to VEP maturation.

**FIGURE 2:**
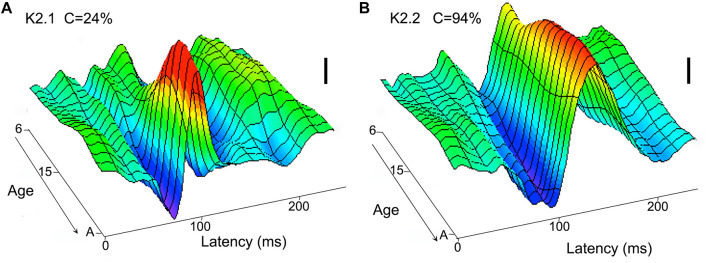
Neurotypical age development (from 6 year to adult) of visual cortical second order non-linearities. The first slice (K2.1) of the second order kernel **(A)** is dominated by M pathway input and shows a maturation at around 8–10 years, while the second slice (K2.2) of the second order kernel **(B)** shows a relatively mature waveform in the youngest participants (6 year). The scale bars represent 100 nV. Figure redrawn from Crewther et al. (1999).

Crewther et al. (1999) used multifocal flash VEP to study the development of non-linear components generated by the M and P systems in children aged between 6 years old and young adult. They found that while a mature form of the P-generated waveform is already apparent at 6 years of age (K2.2, see Figure 2B), the M waveform remain small in amplitude and does not appear to mature until 8–10 years (K2.1, see Figure 2A).

Interestingly, evidence for an early adolescent maturation of motion processing comes from several sources. Langrova et al. (2006) showed the presence of an early positivity in the motion onset VEP at a latency of around 200 ms that was clearly present in a group of 6–7 years children, still discernible at age 12, but had vanished beyond about 16 year age. More recently, Manning et al. (2019) showed, using high density EEG recordings of onset to coherent dot motion in neurotypical development from 6 year to adult, that the younger children showed an extra contribution over lateral occipital cortex that was no longer present by ages 10–12 year.

### Neuropsychology of Early V1 Lesions

Developmental information can be gained from childhood lesion studies. Thus, the case of patient BI - who, at 9 days old, suffered from MCAD a syndrome characterised by seizures and neonatal glycemia is of interest. At 4.5 years a routine MRI scan revealed that BI had extensive occipital grey and white matter loss. Between 6 and 7 years old BI underwent a range of visual tests, showing visual conscious awareness in both dorsal and ventral stream-related function. MRI-based diffusion scanning (Mundinano et al., 2019a) showed similar connectivity patterns to neonatal marmoset cortical lesion studies (Warner et al., 2015). Children with later occipital lesions, such as patient GY, with unilateral left occipital lobe trauma-based damage at the age of 8 years old, tend to show blindsight capability, but not conscious visual perception. The neuropsychological cases here are consistent with a tapering of neuroplasticity by the age of 8 years.

### Emotional Attention

The role of the amygdala in rapid alerting to unusual or threatening events, *via* the so-called “low road” (Pessoa and Adolphs, 2010): retina – superior colliculus – pulvinar – amygdala is well-accepted. A recent paper using orthograde and retrograde tracing in the macaque shows a route for rapid SC inputs to the amygdala by colocalisation of terminals in the medial pulvinar (PM) particularly (Elorette et al., 2018). Similarly, in humans, density of white matter tracts from pulvinar to amygdala are predictive of fearful face recognition (McFadyen, 2019).

However, to date, the means by which threat information thus analysed is seamlessly fed into the transient visual attention system is less well known. It is clear that an emotive stimulus, such as viewing the image of someone pointing a gun in your direction, causes one to attend without thinking – more rapidly than conscious identification of the object as a weapon. One likely mechanism is a specialised projection from the amygdala to the thalamic reticular nucleus (TRN), a thin sheet of cells that wraps around the thalamus, including pulvinar and LGN (Zikopoulos and Barbas, 2012; Halassa et al., 2014; John et al., 2018). Such projections can cause response gain modulation of sensory processing, as shown by optogenetic manipulations in the auditory system of rodents (Aizenberg et al., 2019). Optogenetic activation of basolateral amygdala resulted in large increases in tone-evoked auditory cortical activity, suggesting that stimulation of the TRN by the basolateral amygdala primes thalamus neurons to promote relevant sensory input. If these findings translate to human, such gain in modulation would enhance a response to threat as seemingly higher contrast and hence facilitating the grabbing of attention.

The role of the pulvinar in manipulating sensory response gain is also supported by physiological/pharmacological investigations. Purushothaman et al. (2012) carried out two pharmacological experiments, one inactivating the lateral pulvinar (PL) with muscimol - resulting in a strong diminution of cortical but not LGN visual responses. The second approach created focal PL excitation with the GABA_*A*_ receptor antagonist bicuculline methiodide (BMI). This resulted in a dramatic fourfold increase in visual responses in the supragranular layers of area V1. Thus, a critical role of the pulvinar in gating visual responses is apparent.

## Update - Abnormal Dorsal Stream Development and Connectivity in Autism

### Early Dorsal Stream Deficits in Autism?

When reviewing which parts of the neural substrate might be functioning differently in autism, Dakin and Frith (2005) considered the abnormalities involving coherent motion processing, acknowledging that the magnocellular/dorsal visual pathways would bear a large proportion of these perceptual disturbances, and as indicated above argued that sensory processing of motion in early cortex was probably not the initial neural site of divergence, but that areas in extrastriate cortex, most notably pSTS should be a central focus.

However, the availability of non-linear VEP recordings (Klistorner et al., 1997), at least in comparisons of those with high versus low AQ scores (Sutherland and Crewther, 2010; Jackson et al., 2013; Brown and Crewther, 2017; Mu and Crewther, 2020) allows for a re-examination of the initial site of anomalous dorsal stream function. It is clear from the non-linear analysis of the VEP that there is no evidence of latency effects indicative of impaired afferent information flow of either the M or P pathways. However, in terms of temporal structure –those with high AQ scores show significantly higher amplitude K2.1 (second order, first slice) responses. MEG recordings using similar binary pseudo-random stimuli indicate contributions to the first and second order kernel responses come from primary visual cortex (V1), from area MT+, as well as from other extrastriate cortical sites (presumably area V3a) (Crewther et al., 2016). Thus, non-linear analysis points to early cortical abnormalities at the initial stages of magnocellular processing. Furthermore, the increased second order amplitude is reflective of an impairment in neural efficiency. Also, this impaired neural efficiency occurring in those with ASD or high AQ correlates with lowered flicker fusion thresholds (Thompson et al., 2015; Brown et al., 2018). This brings the likely site of initial M-pathway dysfunction earlier than STS and probably sited in MT+ or possibly in V1. Additionally, arguments of normal magnocellular function based on lack of difference in flicker contrast sensitivity between those with ASD and TD groups (Pellicano et al., 2005) were based on experiments carried out at temporal frequencies (∼10 Hz) that are unlikely to challenge the temporal capability of the M neurons. In reviewing neuroimaging studies in autism, with multiple affected areas related to visual perception, Chung and Son (2020) concluded that abnormalities are not only present in early visual processing but are specifically atypical in the primary visual and extra-striate cortex including area MT.

### Autistic Vision – M-Pathway Dysfunction Alone?

The emergence of non-linear VEP resulted in the ability to directly interrogate magnocellular processing as a function of autistic tendency. Both Sutherland and Crewther (2010) and Jackson et al. (2013) identified a lesser ability to recover after high temporal frequency stimulation for those with high autistic traits. This is a logical interpretation of a significantly larger amplitude of the second order K2.1 kernel responses. However, in addition, Sutherland and Crewther (2010) noted in recording non-linear VEP from a group with high AQ scores, an anomaly that they reported as a delay in the K2.1 peak recorded at high contrast. The authors indicated that the deviant waveform did not fit any of the known contrast responses and hence the neural signature was “foreign” to some extent. This high contrast, long latency anomalous peak was also present in the recordings of Jackson et al. (2013). Close inspection of the low and high contrast recordings of the K2.1 kernel contribution of Sutherland and Crewther (2010) shows a waveform deviation at around 72 ms latency of the high versus low AQ groups. There is a small but visible deviation for low (24% luminance contrast recordings) but becomes dominant at high contrast (96%). Viewed as the contribution of a separate peak, it could be described as a negative-positive N90-P120 peak showing a lack of contrast saturation and possessing a peak latency approximately 25 ms greater than the M contribution. Thus, it does not resemble the M-pathway contribution to K2.1 first recognised by Klistorner et al. (1997), neither does it contribute to the K2.2 kernel response, dominated by the P-pathway.

### Paradoxical Motion Performance in Autism Spectrum Disorders

In addition to a proposed inefficient magnocellular system and more efficient parvocellular system, differences in neuronal properties may also account for the disturbances observed in autism. Investigating the centre-surround properties of motion related stimuli in typically developed individuals led to the concept of “motion surround suppression”; an increased difficulty to perceive stimulus motion direction under conditions of short temporal duration, as stimulus size is increased at high contrasts (Tadin et al., 2003). Surround suppression is believed to reflect centre-surround inhibition, demonstrated in primate area MT single cells (Born and Tootell, 1992; Born and Bradley, 2005) and is suspected to be abnormal in those with autism. At high contrasts, autistic children (mean age 12 years) were found to have a twofold performance increase when compared to their low-contrast results revealing a lesser spatial suppression pattern and an abnormal gain (excitatory/inhibitory) imbalance (Foss-Feig et al., 2013). Note that these differences were found particularly for high contrast stimuli, though see Schauder et al. (2017). This response is interesting because of the expected amplitude saturation for a high contrast, magnocellularly driven mechanism (Kaplan et al., 1990).

Interestingly, in autistic adults, larger population receptive fields derived from fMRI studies were found in area MT+ (Schwarzkopf et al., 2014). The authors suggested that this might be due to extra-striate hyperexcitability, however, the literature on magnetic resonance spectroscopy (MRS) assay of excitatory neurotransmitters such as glutamate as compared with inhibitory transmitters (typically GABA) is rather mixed [compare Robertson et al. (2016) and Ford et al. (2017)]. Schauder et al. (2017) also suggested that those with autism consist of larger excitatory receptive fields on the basis of their findings. Moreover, neurotypical infants over the age of 6 months old showed higher sensitivity to smaller moving objects similar to adults while infants under 6 months had higher sensitivity to larger patterns (Nakashima et al., 2019). The results of larger receptive fields and altered gain responses of MT that show deficiency to small moving stimuli; a mechanism that is usually developed after 6 months in infants could be reflective of altered developmental processes in autism during that period.

### Altered MT+ Afferents in Autism - A Novel Hypothesis

Evidence or larger receptive fields in area MT, together with the developmental knowledge gained from the marmoset (Bridge et al., 2016) leads to a simple but compelling model for developmental changes in neuroanatomy corresponding to variation in autistic tendency. We propose that the post-natal withdrawal of the direct PI_*m*_ → MT pathway in human occurs at a reduced rate in a manner associated with the degree of autistic tendency. Any competition between PUL and V1 inputs to MT would normally be won by the overwhelming numerical advantage of the latter as its later-developing synapses take hold. However, hyperactivity of the PI_*m*_ → MT projection may slow that process, particularly if one considers the response gain characteristics of Pulvinar excitation (Purushothaman et al., 2012). As well as causing fewer fibre connections to be lost, such a slowing would likely result in a longer time to maturation of the MT afferents. Observations involving human MT+ have shown structural and functional abnormalities across a range of ages in those with autism. In their meta-analysis Nickl-Jockschat et al. (2012) found that morphological abnormalities of MT were present in autistic individuals aged from ∼9 to 38 years old with grey matter and hence its volume increasing bilaterally up until puberty but reducing in the oldest cohorts. Nickl-Jockschat et al. (2014) further showed in their activation likelihood estimation meta-analysis that structural deficits of MT were unique to the right hemisphere portion of the area MT in addition to hypoactivation of the left fusiform gyrus during face processing. In terms of motion and M pathway processing, as discussed above, it appears that maturation occurs at ages around 10 years (Crewther et al., 1999; Langrova et al., 2006; Manning et al., 2019). Thus, we are suggesting that maturation in the case of high autistic tendency leaves a higher remnant PIm → MT projection in the mature visual system.

In comparison to neurotypicals, some studies involving young autistic adults have shown stronger MT activation to passive motion (Takarae et al., 2014) as well gamma band power for which an autistic group showed increased activation as a function of the percentage of coherently moving dots. Others (Herrington et al., 2007) have found reduced MT+ activation in Asperger’s syndrome, at least for biological motion stimuli (point light walkers), but not for the control randomly moving dots.

When comparing MT and V1 activity in imaging studies, individuals with ASD tend to show increased MT activation but mixed V1 activity. In an adolescent autistic group (aged 13–19 years) significantly increased MT activation was found in the left MT during random motion with significantly higher left V1 activation (Brieber et al., 2010). In an older autistic group (15–27 years), Robertson et al. (2014) found that for coherent stimuli lasting ∼0.6 s, both MT and V1 had greater responses for an autistic group compared to neurotypicals. Alternatively, a comparison of 18–31 years old autistic and control groups showed that although autistic MT+ activation was significantly higher, their V1 responses were attenuated (Kolodny et al., 2020). Additionally, the authors showed that this MT+ amplification/V1 attenuation was associated with autism severity although only when MT+ was being strongly driven, both at high luminance contrast and high percent coherence. Conversely, other studies have shown significantly less activity in MT for biological motion (point light walker) stimuli in young adults (Herrington et al., 2007) or similar MT+ activity for adolescents (Koldewyn et al., 2011) when compared to neurotypicals.

### The Amygdala and Anxiety in the Developing Autistic Brain

Since Kanner in 1944 and Tinbergen in 1976, many have argued that fear, anxiety or apprehension dominates the behaviour of the infant that becomes an autistic child. This has brought focus on the anatomy and connectivity of the amygdala due to its role in emotional processing, as well as links to social cognition and salient attention (Davis and Whalen, 2001; Adolphs and Spezio, 2006; Adolphs, 2010). This hyperactivity of the amygdala was suggested to lead to suppression of normal development of social behaviour (Tinbergen and Tinbergen, 1976) hence warranting further investigation of amygdala morphology and function. Amygdalar volumes as well as amygdalar neuron numbers in autistic *vs.* control children (6.5–12.0 years) demonstrate early relative increases (Avino et al., 2018; Xu et al., 2020) that dissipate by adolescence (13–19 years). By early adulthood (20–27 years), the early increase in amygdala volume and neuron numbers has been reversed. Some of these differences have been associated with the level of anxiety and stress in the autistic populations (Nacewicz et al., 2006). Anxiety is a common symptom in those with ASD and the broader autism phenotype (BAP) (Hallett et al., 2013). Functionally, developmental deficits involving the amygdala and down-stream social and emotional processing cortical areas have been proposed to contribute to behaviours in ASD (Schultz, 2005).

In autism, however, the amygdala appears to exhibit a period of early enlargement found in young children specifically around the ages of 2–4 years (Sparks et al., 2002; Bellani et al., 2013) but not in adolescents (Schumann et al., 2004). Slightly older autistic children with a mean age of 11 years old were found to have decreased right amygdala volumes (Herrington et al., 2017) while those aged from 13 to 19 years had no amygdala change when compared to neurotypicals (Xu et al., 2020). Moreover, Mosconi et al. (2009) who used a longitudinal design found that reduced amygdala volume is found earlier at the age of 2 years but not between the ages of 2–4 years in autism; also highlighting that larger amygdala is associated with a greater facility for joint attention. This pattern of early enlargement and later reduction was also found in deceased autistic individuals aged from 2 to 48 years where amygdala neuron population was initially in excess but reduced during adolescence and adulthood (Avino et al., 2018).

We further propose that maintaining consistent activation of this low-road pathway in autism, particularly *via* hyperactivity of the PUL → amygdala projection will diminish the pruning of the early PUL → hMT connections especially during very early development. Interestingly, both the amygdala and pulvinar have been found to modulate incoming salient information in neurotypicals. Increased (left) amygdala activation was found to interfere with motion perception and decrease motion related activity in area MT (Hindi Attar et al., 2010) and optimise dynamic and static fear responses in dorsal (MT) and ventral (FFA) regions, respectively (Furl et al., 2013). While some have suggested weak amygdala modulation in autism (Sato et al., 2020) others have found increased pulvinar to (right) amygdala connectivity in adolescents and that this connectivity is correlated with sensory over-responsivity severity (Green et al., 2017). Moreover, the amygdala shares direct connections with the thalamic reticular nucleus (TRN), a hub crucial for the control of gating of thalamocortical signals. This amygdala → TRN projection is suggested to rapidly shift our attention to emotional stimuli (Zikopoulos and Barbas, 2012) and may be further implicated in autism (Zikopoulos and Barbas, 2012; Krol et al., 2018). Hyper-connectivity of pulvinar, specifically the medial pulvinar has been found in autism from the thalamus (Woodward et al., 2017) and has been suggested to contribute to modulation of response gain in a range of neurodevelopmental disorders, including autism (Homman-Ludiye and Bourne, 2019). Additionally, overmodulation of the pulvinar in ASD has been found to reduce MT+ activations as well as to predict increased clinical symptoms (Martinez et al., 2019).

### Autism - Fragility of Binocular Function?

Simmons et al. (2009) suggested that the unusual sensory processing in autism is at least concomitant and possibly the cause of many of the behavioural signs and symptoms. Simmons also pointed to weaker binocular visual function in autism noting an abnormally high incidence of strabismus in autistic cohorts, as well as abnormalities in visual pursuit and optokinetic nystagmus (OKN). Further analysis of the literature indicates a mean incidence of strabismus of approximately 20% (range 10.0–50%) in those with a clinical diagnosis of ASD (Scharre and Creedon, 1992; Kaplan et al., 1999; Ikeda et al., 2013; Wang et al., 2018; Khanna et al., 2020; Chang et al., 2021), compared with mean normal incidence of <3%. Curiously, such a fragility of binocular function might be related to the reported reduction of motion surround suppression in children with ASD compared with controls (Foss-Feig et al., 2013). Even within TD populations the lack of binocular engagement, as occurs when viewing monocularly, also results in reduced motion surround suppression (Arranz-Paraiso et al., 2021). Given the contralateral nature of the retina → PIm → MT projection, it is likely that an enhancement in this projection in those with high levels of autistic tendency would weaken the strength of binocular function.

### Late Developmental Maturation of Extrastriate Connections in Autism Spectrum Disorders

We have hypothesised that childhood disruptions related to autistic tendency occur in the trajectory of visual system development and involve altered thalamic circuity to MT+. If a reduced withdrawal of thalamic (PUL → MT) projections occurs, then this would likely commence in children of ages 0–2 years as area V1 → area MT projections develop. Brain volume overgrowth is observed in the 12–24 month period coinciding with the emergence and severity of autistic social deficits (Hazlett et al., 2017) as well as the regressive type of autism (Nordahl et al., 2011). However, visual perception and functional neuroimaging of the visual system has been confined to later in childhood development. Diffusion weighted MRI studies comparing those with autism against neurotypicals across the developmental span suggest that childhood group differences in microstructure of the thalamus and posterior limb of the internal capsule (PLIC) become less robust in adolescence and adulthood (McLaughlin et al., 2018). Indeed, a childhood divergence of tract development is reported, with the ASD group showing increased mean diffusivity, radial diffusivity, and axial diffusivity in the posterior corpus callosum. Resolution of such differences were found in children at approximately 10 years of age (Travers et al., 2015), a time frame very similar to that of Wiener kernel maturation of the visual magnocellular VEP (Crewther et al., 1999). It is possible during this extended period that the functional neural circuit relating form and motion processing may develop along different trajectories. To this end, McKay et al. (2012) suggested, using a Granger causality approach in fMRI, that biological motion is handled by a different pathway circuit in those with ASD compared with TD individuals. While the TD group activated a unitary circuit integrating form and motion regions in interpreting biological motion stimuli, the ASD group, by contrast, appeared to use two independent circuits, one based around form while the other was based around motion sensitive areas. The presence of these dissociated circuits may underlie the apparent antagonistic activations of V1 and MT seen for ASD but not TD processing (Kolodny et al., 2020). Pitcher and Ungerleider (2021) have recently suggested that the involvement of moving stimuli plays a fundamental role in a third visual pathway specifically for social perception, extending from V1 → MT → pSTS → anterior STS (aSTS). Additionally, a cortico-amygdala face processing branch has been shown to exist *via* the right pSTS → anterior STS → amygdala (Lahnakoski et al., 2012; Pitcher et al., 2017). Considering the strong feedforward connections between MT+ and STS and consistent deficits of the pSTS in relation to biological motion observed in autism, late or altered developmental maturation of this social pathway is also likely present.

### Discussion and Outstanding Questions

This review has placed findings coming from primate development, particularly the marmoset in terms of developmental plasticity, the importance of initial pulvinar projections from the inferior Pulvinar to Area MT for the establishment of the dorsal cortical stream alongside new understanding of dorsal stream perceptual and physiological differences in humans with high versus low autistic tendency. Several lessons are immediately obvious – the projections from PIm → MT are not magnocellular, in the sense that they are not driven by the parasol type of retinal ganglion cell. The implications of another class of ganglion cell setting up the dorsal stream prior to final domination of MT by M pathway projections *via* the LGN are diverse. The large receptive field size of smooth wide-field ganglion cells would predict a developmental reduction in human population receptive field size with age. A postulated reduction in withdrawal of PIm → MT projections in ASD also conforms with the finding of increased pRF size in ASD compared with controls. Further, the contrast response properties of such cells indicate lesser surround suppression and a tendency for motion integration between receptive fields, as one would find for background motion processing. The high contrast non-linear VEP anomalies recorded in those with high autistic tendency are supportive of contribution from an additional cell class to these second order kernels, apart from the well-recognised magnocellular class.

Furthermore, the proposed aetiology of autism might relate to other neurodevelopmental conditions such as dyslexia. Not only do dyslexics show decreased motion performance – suggested to be driven by magnocellular/dorsal stream impairments (Livingstone et al., 1991; Stein, 2019; Brown et al., 2020; Peters et al., 2020) but also show altered/reduced structural connections between the left visual thalamus and left area MT, suggesting a deficient left-hemispheric motion processing system (Muller-Axt et al., 2017; Rima et al., 2020).

This review has not attempted to cover the diversity of genetic associations with autism (Satterstrom et al., 2020). However, one is tempted to pursue the question of gene associations from the other direction and ask whether there are candidate genes that control post-natal neurodevelopmental change that result in altered neural circuitry. Alteration within the MET receptor tyrosine kinase may be a critical contributing genetic factor in autism, with variable disruption of post-natal neurological plasticity, depending on cell context (Eagleson et al., 2017). Curiously, prolonging MET signalling for 2 weeks in developing mice results in repetitive actions and social impairment (Ma et al., 2021).

An idea proposed often leaves many unanswered questions:

1 How does human post-natal neurodevelopment of pulvinar → MT compare with that in the marmoset?
2 Can diffusion tractography identify differences in input from thalamus to Area MT in those high and low in autistic tendency?
3 Does the attentional field extent exhibited by those with high versus low autistic tendency relate to global versus local perceptual preference?
4 How does early development of MT impact STS development and social function in those with high versus low autistic traits?
5 How do other syndromes exhibiting dorsal stream vulnerabilities, such as dyslexia, relate to ASD in terms of neural dysfunction?

## Conclusion

The past 20 years has allowed for greater reflection on the developmental processes at play in those with autism and high autistic tendency. While the role and deficits attributed to the STS for socially relevant moving stimuli is undeniable this review has presented accompanying evidence suggesting that the fundamental driver of individual differences along the autism spectrum lies in the connectivity and processing of area MT+. A theory based on early restricted withdrawal of the inferior pulvinar to MT connections results in much larger receptive field motion sensitive inputs and concurs with observations of larger population receptive field size for area MT in autism.

Such developmental slowing of PIm → MT fibre withdrawal, associated with raised amygdala activity early in life is proposed to act *via* the amygdala → TRN gatekeeper control of the pulvinar that enhances visual responsiveness. Larger MT population receptive field size (Schwarzkopf et al., 2014) is likely to help explain the paradoxical enhancement of motion perception when investigating the temporal properties of motion perception in autism. The presence of an enhanced monocular input may also explain the relative fragility and high incidence of strabismus in those with ASD.

## Author Contributions

SS reviewed the available literature and wrote the drafts of the manuscript. Both authors contributed equally to editing the manuscript.

## Conflict of Interest

The authors declare that the research was conducted in the absence of any commercial or financial relationships that could be construed as a potential conflict of interest.

## Publisher’s Note

All claims expressed in this article are solely those of the authors and do not necessarily represent those of their affiliated organizations, or those of the publisher, the editors and the reviewers. Any product that may be evaluated in this article, or claim that may be made by its manufacturer, is not guaranteed or endorsed by the publisher.
